# Direct Detection of Soil-Bound Prions

**DOI:** 10.1371/journal.pone.0001069

**Published:** 2007-10-24

**Authors:** Sacha Genovesi, Liviana Leita, Paolo Sequi, Igino Andrighetto, M. Catia Sorgato, Alessandro Bertoli

**Affiliations:** 1 Dipartimento di Chimica Biologica, Università di Padova, Padova, Italy; 2 Istituto Sperimentale per la Nutrizione delle Piante, Gorizia, Italy; 3 Istituto Sperimentale per la Nutrizione delle Piante, Roma, Italy; 4 Istituto Zooprofilattico Sperimentale delle Venezie, Legnaro, Italy; 5 CNR Istituto di Neuroscienze, Padova, Italy; Université de Toulouse, France

## Abstract

Scrapie and chronic wasting disease are contagious prion diseases affecting sheep and cervids, respectively. Studies have indicated that horizontal transmission is important in sustaining these epidemics, and that environmental contamination plays an important role in this. In the perspective of detecting prions in soil samples from the field by more direct methods than animal-based bioassays, we have developed a novel immuno-based approach that visualises *in situ* the major component (PrP^Sc^) of prions sorbed onto agricultural soil particles. Importantly, the protocol needs no extraction of the protein from soil. Using a cell-based assay of infectivity, we also report that samples of agricultural soil, or quartz sand, acquire prion infectivity after exposure to whole brain homogenates from prion-infected mice. Our data provide further support to the notion that prion-exposed soils retain infectivity, as recently determined in Syrian hamsters intracerebrally or orally challanged with contaminated soils. The cell approach of the potential infectivity of contaminated soil is faster and cheaper than classical animal-based bioassays. Although it suffers from limitations, e.g. it can currently test only a few mouse prion strains, the cell model can nevertheless be applied in its present form to understand how soil composition influences infectivity, and to test prion-inactivating procedures.

## Introduction

Prions are infectious pathogens causing fatal neurodegenerative disorders, known as transmissible spongiform encephalopathies (TSEs), or prion diseases, which affect different mammalian species. TSEs include scrapie in sheep, bovine spongiform encephalopathy (BSE) in cattle, chronic wasting disease (CWD) in mule deer, elk, and moose (cervids), and Creutzfeldt-Jakob disease (CJD) in humans. The prominent, if not only, component of prions is a misfolded conformer (PrP^Sc^) of a constitutive sialoglycoprotein, the cellular prion protein (PrP^C^) [1].

In contrast to the inability of scrapie prions to cross the ovine-man species barrier, BSE prions can be transmitted to humans through the consumption of infected beef products, giving rise to the novel human prion disease, named variant CJD (vCJD) [2]. As for CWD, the risk of transmission to humans is currently unknown, but the recent, extensive spread of the disease among free-ranging cervids in some U. S. areas raises concerns for public health [3], [4]. Recently, a statistical analysis has shown no significant increase in the risk for CJD in areas with high CWD prevalence. However, the still unknown clinical features of CWD in humans does not yet allow to definitively discard the possibility that CWD prions cause human disease [5]. A notable feature of scrapie and CWD is horizontal transmission between grazing animals [6]–[9], implying that contaminated soil may serve to propagate the disease. In this respect, it has been reported that grazing animals ingest from tens to hundreds grams of soil per day, either incidentally through the diet, or deliberately in answering salt needs [10], [11], and that mule deer can develop CWD after grazing in locations that previously housed infected animals [12]. Prions may enter the environment through different routes, including animal's excreta and secreta [12]–[14]. Using different techniques, several laboratories, including ours, have shown that recombinant (r) PrP [15]–[19] and brain PrP^C^ and PrP^Sc^ [20]–[24] strongly bind to different types of soil and soil mineral, but also that soil mineral-sorbed prions can transmit disease when intracerebrally inoculated [21]. This fact, together with the unusual resistance to degradation by conventional agents of pathogenic PrP^Sc^, supports the notion that prions may persist for long periods of time in soil [24], [25], thereby increasing the probability of intraspecies or interspecies transmission. Indeed, after persisting in soil for more than two years, a hamster-adapted prion strain was shown to retain pathogenic activity, and transmit disease in Syrian hamsters via the oral route [24] (see also [26]).

In view of the prion-related risk in the environment, which calls for the prompt identification of prion-contaminated soils, we present here alternative protocols that, on the one hand, detect *in situ* PrP^Sc^ bound to agricultural soil samples; on the other hand, may allow a more rapid assessment of the infectious potentials of soils than animal-based bioassays.

## Results and Discussion

### PrP^C^ or PrP^Sc^ Bound to Soil Can Be Visualised Directly by Immunodetection

Previously, the avid binding of both PrP^C^ and PrP^Sc^ to soils/soil components [20]-[22], [24], and the capacity of PrP-soil interactions to withstand harsh conditions, including boiling in denaturing detergents [20] (see also [21]), have been either inferred from the loss of PrP^C^/PrP^Sc^ immunoreactivity in the liquid phase (following centrifugation of soils treated with brain homogenates) [20], or demonstrated after extraction from soils [21], [22], [24]. We thus thought it valuable to find means by which to assess soil-bound prions in a direct way, without need of desorbing PrP^Sc^ prior to detection procedures. Inspired by the assay for the binding of prions to steel surfaces [27], we developed an *in situ* assay, whereby samples of arable sandy-loam (ASL)-incubated (for 1 h) with diseased brain homogenates-are probed with anti-PrP antibody to visualise directly the major component of prions, PrP^Sc^, sorbed onto soil particles.

**Figure 1:**
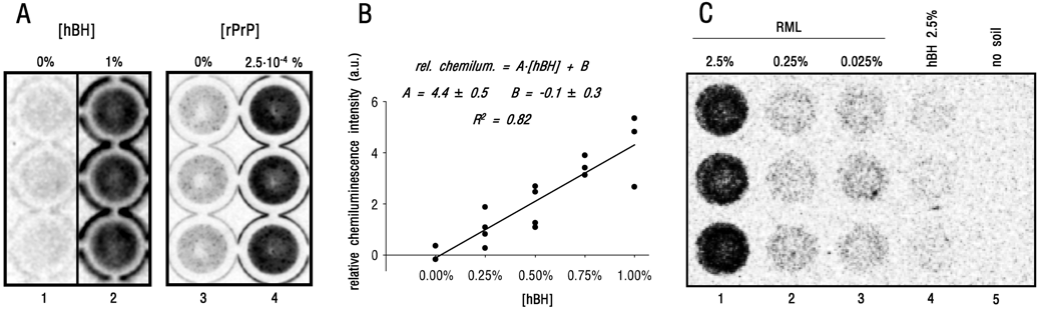
Direct Visualisation of Soil-bound PrP^C^ and PrP^Sc^. (A) Samples (100 mg) of arable sandy loam (ASL) are incubated (1 h, RT; final volume, 200 µl of PBS) in the absence (column 1), or in the presence (1% (w/v), column 2), of healthy mouse brain homogenate (hBH). After sedimentation (30 g, 5 min), collected soil particles are extensively washed, and the presence of soil-bound PrP^C^ immunodetected as described (see Materials and Methods). The same protocol is used for the incubation of ASL in the absence (column 3), or in the presence (column 4), of the indicated concentration (w/v) of recombinant murine PrP (rPrP). Soil-bound immunoreactivity appears as a dark chemiluminescent signal, clearly distinguishable from the background (i.e. observed in the absence of PrP). (B) A titration experiment is reported, in which progressively increasing PrP^C^ quantities are incubated (1 h, RT) with ASL, under conditions of constant total tissue-to-soil ratio. This is achieved by mixing different volume ratios of hBHs (10% (w/v) in PBS) from wild-type (WT) and PrP-knockout (KO) mice (WT∶KO (µl∶µl) 0∶20, 5∶15, 10∶10, 15∶5, 20∶0) (final volume, 200 µl of PBS). The resulting final concentrations (w/v) of WT brain homogenates (hBH) are as indicated. The relative amounts of immunodetected soil-bound PrP^C^s, determined by the densitometric analysis of the collected chemiluminescence, subtracted for the background signal ([hBH] = 0%), are reported (in arbitrary units, a.u.) in the scatter plot as a function of [hBH]. Regression analysis indicates a linear relation (with a p value<0.05 (t-Student test)), at least within the tested concentration range, and demonstrates that the assay is specific for PrP^C^. Reported are also the regression line, the regression equation (with the standard error for the slope and the intercept) and the R^2^ value. Other experimental details are as in (A). (C) To visualise soil-bound prions (columns 1–3), ASL samples are incubated with the indicated final concentrations ((w/v) in PBS) of RML-infected mouse brain homogenates (serially diluted into non-infected (2.5%) homogenates), before applying to purified soil pellets the above described immuno-based procedure, implemented with PK digestion and addition of guanidinium thiocyanate (see Materials and Methods). The obtained dark immunosignal, readily appreciable with respect to soil samples incubated with hBH (2.5%, column 4), is therefore indicative for the presence of PK-resistant soil-bound PrP^Sc^. The null response, obtained by processing RML-brain homogenates (2.5%) in the absence of soil (no soil, column 5), demonstrates that the signal is specific for PrP^Sc^ adsorbed onto soil surfaces, rather than resulting from (contaminating) not bound PrP^Sc^. Other experimental details are as in (B).

To set up the protocol, at first we tested healthy brain homogenates (hBH) (i.e. PrP^C^). As shown (Fig. 1A, column 2), the assay gave in a readily detectable signal for the presence of soil-bound PrP^C^. Given that no immunosignal was detected in soil samples not treated with brain homogenate (column 1), we can conclude that signal was not due to non-specific sorption of the antibody to soil particles. In contrast, a positive response was present in soils incubated with recombinant PrP (rPrP) (cf. columns 3 and 4). We also tested whether the assay could correctly monitor increasing soil-bound PrP^C^s. Indeed, we found a linear relation between signal and concentration of PrP^C^ (scatter plot of Fig. 1B). However, because experiments were carried out using hBH serially diluted with brains lacking PrP^C^, Fig. 1B also indicates that (i) data cannot be attributable to soil-sorbed proteins other than PrP^C^, so it is PrP^C^-specific; (ii) the assay allows appreciation of differences in tissue concentrations as low as 0.25% (w/v).

We then verified the reliability of the assay applied to soil-bound prions, after implementing the protocol with additional steps, including digestion of PrP^C^ by proteinase K (PK). Following this paradigm, an unambiguous signal was found for ASL particles incubated with concentrated (2.5%) brain homogenates infected with the mouse-adapted RML (Rocky Mountain Laboratories) prion strain [28] (Fig. 1C, column 1). Significantly, also 10 and 100 fold-diluted infected tissues were able to provide a higher chemiluminescence than that observed by replacing RML- with healthy-brain homogenates (cf. columns 2 and 3 with column 4). We found no signal with RML-brains subjected to the entire protocol in the absence of soil (column 5), so we excluded the possibility of positive signal due to contamination of the examined sedimented fractions (hereafter also named pellets) (see Materials and Methods) by PrP^Sc^ not bound to soil. Collectively, these results strongly indicate that a PK-resistant PrP isoform, generally considered a reliable marker for the presence of the infectious PrP^Sc^ agent, is sorbed onto ASL particles, and that its interactions with soil withstand the harsher steps of the immunodetection procedure, i.e. exposure to PK and guanidinium thiocyanate (see Materials and Methods).

In conclusion, for the first time to our knowledge, we have developed an immuno-based model enabling the direct identification of PrP^Sc^ bound to soil. Given the ultimate aim of detecting environmentally disseminated prions, the test is particularly interesting as it avoids the separation of PrP^Sc^ from the soil. As yet, however, we have been unable to obtain a reliable quantitative assessment of soil-bound PrP^Sc^. Possibly, this depends on the incubation step with PK that, in addition to degrading PrP^C^, may also detach PrP^Sc^ [22] in a manner that we did not quantify precisely. The fact that ours are solid specimens may, however, pose conceptual obstacles to the possibility of comparing the properties of the presented protocol with those of other immunological procedures that assay PrP^Sc^ in the liquid phase (e.g. Western blot and Elisa). Nonetheless, already in its present form, or after suitable improvements (e.g. optimisation of steps specific for PrP^Sc^ visualisation), the assay may well be valuable for different experimental instances. For example, with respect to the partial recovery of soil/soil minerals-bound PrP^Sc^ after extraction procedures [21], [22], [26], the test could demonstrate whether missing PrP^Sc^ still adheres to soil rather than being degraded, or lost. Another instance regards the strong decrease in PrP^Sc^ amounts that can be recovered from soil over an incubation period of several months. Here also, application of our procedure may help discriminating if this event results from degradation of PrP^Sc^, or from a tighter binding to soil over time, as hypothesised [24].

### Soil-Sorbed Prions Are Infectious

Next, we examined the infective potential of soil-bound PrP^Sc^. To this end, soils were contaminated with RML-brain homogenates. Although PK-resistance is widely accepted as a standard operational rule for prion identification, the decisive proof of infectivity is the biological assay, based on the appearance of TSE-recapitulating neurologic syndromes in animals inoculated with alleged prion-tainted materials. However, strategies employing “scrapie-susceptible” cells have been proposed [29] to circumvent some practical drawbacks of the animal-based approach, e.g. the long incubation periods and elevated costs, as well as risks inherent to inoculating brains with solid, large-sized material (e.g. whole soil). By way of example, the capacity of the clonal murine neuroblastoma N2a-PK1 cell line to sustain propagation of added PrP^Sc^ molecules - both horizontally (from one cell to neighbouring cells) and vertically (from mother cells to daughter cells)-ultimately amplifies prions (though originally added in low amounts) up to levels easily detectable by standard immunological approaches [30].

**Figure 2:**
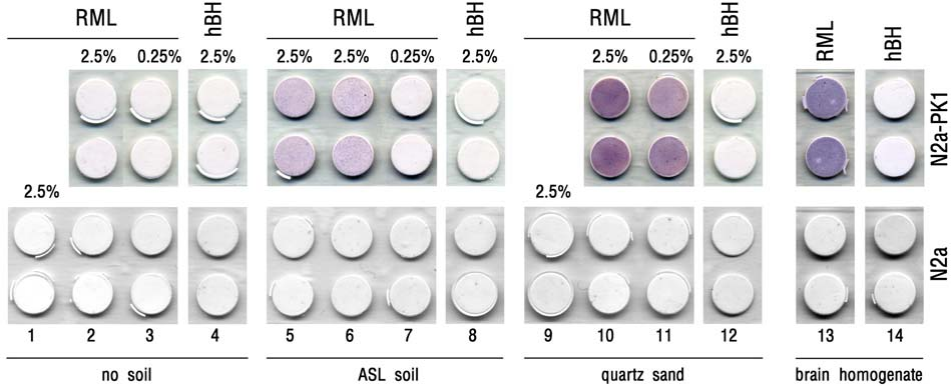
Prion-contaminated ASL and Quartz Sand Can Propagate Prion Infection. Prion-susceptible N2a-PK1 (upper panels), and prion-resistant N2a (lower panels), cells are inoculated with the pellet fraction obtained by centrifuging the indicated concentrations of RML-infected (columns 1–3, 5–7, 9–11), or healthy (columns 4, 8, 12), brain homogenates in the absence (no soil, columns 1–4), or in the presence (2 mg), of (whole) ASL (columns 5–8), or quartz sand (columns 9–12). After 6 (1∶3) and 3 (1∶10) consecutive splittings, cells are analysed for prion propagation using the Elispot assay (6·10^4^ cells/well, run in duplicate). While no signal of infectivity is detectable using hBH (upper panels, columns 4, 8 and 12), a prion-positive immunosignal is evident in N2a-PK1 cells inoculated with ASL samples contaminated by RML-homogenates (2.5%) (columns 5 and 6). Under the same conditions, RML-tainted quartz sand yields a more intense signal (column 10). Also at difference from ASL, a positive result is obtained with quartz particles added with a 0.25% RML-homogenate (cf. columns 7 and 11). The lack of prion amplification by N2a-PK1 cells inoculated with pellets from soil-free RLM-homogenates (columns 2 and 3), indicates that positive signals arise from soil-bound prions, not from (soil-unbound) PrP^Sc^ leftovers in the inoculum. Likewise, incapacity of N2a cells to be infected (lower panels) clearly demonstrates that the anti-PrP immunoreactivity is consequent to the *de novo* production of prions by cells, rather than to residuals of the inoculum in cell culture chambers. Additional controls are the incubation of N2a-PK1, or N2a, cells with a spike of crude RML- (column 13), or healthy- (column 14), homogenate (final concentration 0.025% (w/v)). Shown results are representative of 3 to 6 independent experiments, which always yielded qualitatively comparable results.

As an alternative to animal bioassay as used in previous studies [21], [24], [26], we exploited the properties of this cell-based infectivity assay (scrapie cell assay, SCA) to test preservation of infectivity by prions bound to ASL, or quartz sand. We first excluded contamination of inocula by PrP^Sc^ not bound to soil: soil-free RML-homogenates were processed as described, and the sedimented fraction applied to N2a-PK1 cells. As shown (upper panel of Fig. 2, columns 2–3), under no condition there was a signal indicating infection of cells. Thus, at the low speed utilised to recover soil particles, prions not bound to soil do not sediment in quantities sufficient to be detected by SCA. Instead, infectivity was clearly evident with cells treated with ASL samples incubated with a (2.5%) sick brain homogenate (columns 5 and 6). An important cross control of this finding is the observation that the liquid phase of soil-free RML-brain homogenates became strikingly less infectious after soil addition (see Fig. S1, supplementary information online), indicating that a major amount of prions gets sequestered by soil particles. To note that, in order to avoid contamination of cell cultures by microorganisms, soil material was always subjected to sterilisation by autoclaving before mixing with brain homogenates (see Materials and Methods). It has been reported that this procedure modifies the sorptive properties of soil [31], [32]. In these works, however, soils with different properties than those of ASL, and harsher sterilisation conditions, were used. Autoclaving should favour removal of dissolved organic carbon. This component, however, was present in our ASL sample only in negligible amounts (determined by extraction with 1 mM CaCl_2_, data not shown). In addition, although thermal treatment may alter physical and mechanical properties (cation exchange capacity, compressibility and particle size) of soil's mineral components [33], [34], it apparently does so at much higher temperatures (>300°C) than that used in our protocol (121°C). Finally, at least with regard to the capacity to bind PrP^Sc^, no difference between sterilised and unsterilised ASL was found (data not shown). Nonetheless, in the prospect of assaying field samples by SCA, we cannot exclude that sterilisation results in reduction of infectivity, and, hence, in false negatives. At the same time, however, it is our opinion that also omitting to sterilise field samples may influence the correct quantification of infectivity by the animal-based assay. In this case, one cannot exclude that the presence of inflammogenic pathogens in the inoculum stimulates PrP^C^ expression [35] and, consequently, PrP^Sc^ accumulation.

Intriguingly, using identical incubation conditions, a much stronger signal was observed with pellets of RML-treated quartz sand (column 10) that, again at variance from ASL, yielded a positive response also with a 10-fold diluted RML-homogenate (cf. columns 7 and 11). Various explanations may account for this observation. One is that sorption of prions to quartz is more readily reversible than with ASL - in line with the lower amount of PrP^Sc^ sequestered by quartz than ASL (per unit mass) ([20]; Genovesi and Bertoli, unpublished data) - and that detached prions favour transmissibility in cultured cells. However, given that binding to soil minerals enhances prion transmissibility by the oral route [26], one can also hypothesize that, in our cell paradigm, quartz-sorbed prions act as a better template than ASL-bound prions. We know that agricultural soil and quartz sand have distinct properties, so one, or more, of them may account for either hypothesis. Finally, the size of the used quartz microparticles (<2 µm) enables homogeneous soil-to-cell contacts (observations at the microscope, not shown) that, in turn, may promote a more efficient cell infection.

As expected, a strong response was obtained after incubating N2a-PK1 cells with a spike of crude RML-infected homogenate (column 13). Conversely, no signal was evident when using healthy brain homogenates (columns 4, 8, 12 and 14), or cells incompetent for prion propagation (i.e. the N2a cell line) (Fig. 2, lower panels). The latter data reinforces the notion that positive results are attributable only to prions *de novo* synthesized by N2a-PK1 cells, not to traces of the original inoculum persisting in the culture plates. Importantly, the infection degree of N2a-PK1 cells challenged with prion-contaminated soil/quartz appears to be dose-dependent, at least in the used range of RML-homogenate concentrations (cf. columns 5–7 and 10–11, upper panels). We did not, however, pursue the precise quantitative assessment of infectivity of prion-contaminated soil/quartz by SCA.

Notwithstanding that determination of soil-sorbed prion infectivity by SCA imposes stringent requirements, the specific use in the assay of (also) an arable sandy loam enriched of all its (heat-stable) natural components, and tissue homogenates, aimed at approximating field conditions. This is important, in view of the possible environmental contamination by prions following incautious human practices (e.g. buried carcasses, use of bone meal fertilizers) [36], or by animal remains (e.g. placentae, carcasses) or secreta [37], [38], as also indicated by the presence of prions in the saliva and blood of deer with CWD [14], and in the urine of scrapie-affected sheep suffering from chronic nephritis [13]. Their strong binding to soil components and the conservation in soil for long periods of time [24], [25] suggest that prions persist at the ground surface rather than leach into deeper layers, or be naturally degraded. Clearly, these are conditions facilitating infection of grazing animals through the oral route. Finally, given that PK resistance is maintained by soil-bound PrP^Sc^, and that desorption of prions from soils is unlikely to occur (at least under our used conditions), our data add further support to the hypothesis that prions need not to be detached from soil particles to self-propagate into host cells, and that, when sorbed to soils, they may increase prion transmissibility by avoiding, or delaying, clearance by host organisms (see also [26]).

In the context of assays that could be used to detect infectivity of natural and farm soils, it is important to acknowledge another crucial limitation of the presented cell model of infectivity. Being N2a-PK1 cells susceptible to only some murine prion strains, these cells cannot evaluate contamination by scrapie and CWD prions, the most relevant for environmental infectivity. Yet, our study may stimulate further investigations to identify cell lines specifically susceptible to these prions. Already as such, however, the presented method may have immediate applications, at least *in vitro*, e.g. to asses if and how different soil components retain infectivity, or to compare the effectiveness of prion inactivating procedures, all of which are crucial for protecting human and animal health from the threat posed by environmentally disseminated prions. Finally, despite all mentioned shortcomings, including the acknowledgment that they may not fully substitute the gold standard bioassay in animals, it is good to highlight that cell-based approaches of prion infectivity have the important value of being rapid, handy and inexpensive.

## Materials and Methods

### Soil and quartz samples

As environmental specimens we used an agricultural sandy soil (arable sandy loam, ASL), and white quartz sand (Sigma-Aldrich). ASL, classified as Calcari-Fluvi Cambisol (FAO, 1982; USDA, 1996), was sampled near S. Martino al Tagliamento (North-East of Italy) at a depth of 2–5 cm, air dried and passed through a 2 mm stainless steel sieve. ASL properties, partly reported in [20], were further characterised as follows: 1) predominant mineralogical composition of clay (semi quantitatively determined by X-ray diffraction analysis) [39]: 12% vermiculite, 8% chlorite, 8% kaolinite, 6% illite, and 6% montmorillonite; 2) specific surface area: 10 m^2^/g (measured by N_2_ adsorption, BET method [40]). In spite of comparable results obtained with non-fractionated samples, in some infectivity experiments ASL was size-fractionated by gravity sedimentation (after suspension in water), so as to be better tolerated by cells. Used fractions contained around 90% clays (particle size, <2 µm) and around 10% aggregated amorphous minerals (2–5 µm) (determined by X-ray diffraction analysis). Quartz sand was used after grinding and size-fractionation by gravity sedimentation (particle size, determined by the gravimetric method: <2 µm). For infectivity assays, sterilisation of both specimens (two autoclave cycles, 121°C, 20 min) always preceded the mixing with brain homogenates.

### Brain samples

Brains from terminally sick CD1 mice, infected with the mouse-adapted RML prion strain [28], were kindly provided by Dr. A. Aguzzi (University Hospital of Zurich, Switzerland) and Dr. R. Chiesa (Dulbecco Telethon Institute, Italy). Dr. Aguzzi also provided *Prnp* knockout (PrP^−/−^) mice. Control (not infected) brains were obtained from CD1 or C57BL/6 mice. Murine rPrP 23–230 [41] was a kind gift of Dr. A. Negro (University of Padova, Italy). Brains, homogenized (10% (w/v)) in phosphate-buffered saline (PBS) by repeated passaging through 20G and 22G syringe needles, were clarified by centrifugation (500 g, 20 min) to eliminate particulate material. Supernatants were then sampled and stored (−80°C), and, immediately before use, diluted as described.

The Department of Biological Chemistry, University of Padova, has been acknowledged for the use of mice for experimental purposes (610/24495/SP/1829 issued by the Italian Health Ministry).

### Preparation of soil-brain homogenate samples

To immunodetect soil-bound PrP^C^/PrP^Sc^, we used ASL (100 mg) incubated, by gentle shaking (1h, RT), in the presence, or in the absence, of wild-type, or RML-infected, brain homogenates (final volume, 200 µl of PBS). In titration experiments, the brain samples were diluted into healthy PrP^−/−^-, or control-brain, homogenates, respectively, to maintain constant the total tissue-to-soil ratio (final concentrations are reported in the figures). Soil particles were then recovered by centrifuging (100 g, 5 min) soil-brain mixtures, washed (4 times) with PBS (1 ml), sedimented again, and finally tested for soil-bound PrP^C^/PrP^Sc^ as described below. When needed, rPrP (at the indicated final concentration (w/v)) replaced brain homogenates. To assay soil-associated prion infectivity, either ASL (2 or 10 mg), or white quartz sand (2 mg), was incubated (1h, RT) with the given concentrations of healthy, or RML-infected, brain homogenates (final volume, 20 µl). Before soil addition, RML-homogenates (starting concentration, 2.5% (w/v) in PBS) were diluted using control brain homogenates (2.5% (w/v) in PBS). After centrifugation (100 g, 5 min), supernatants were carefully collected, and soil pellets subjected to the aforementioned wash-cycle.

### Immunodetection of soil-bound PrP^C^/PrP^Sc^

The direct visualisation of PrP^Sc^ bound to soil particles was carried out using a novel immunodetection assay consisting in: (i) Saturation (1 h, RT) of the soil binding sites for proteins with bovine serum albumin (BSA, 5% in PBS). (ii) Incubation (1 h, RT) of soil pellets with PK (Biorad) (50 µg/ml in PBS containing 0.1% BSA, final volume 200 µl), followed by rinsing with PBS (750 µl), and incubation with PMSF (1 mM in PBS) (200 µl, 10 min, RT) to inactivate PK. (iii) Incubation of sedimented soil particles with Tris-guanidinium thiocyanate (200 µl of a 3 M solution, 10 min, RT) to expose PrP epitopes, followed by washing (4 times) with PBS. (iv) Incubation (1 h, RT) with SuperBlock® (Pierce) in Tris-buffered saline (TBS), according to the manufacturer's instructions, to block non-specific antibody binding sites. (v) Incubation (1 h, RT) with anti-PrP monoclonal antibody (mAb) POM-1, targeted to the region between the first and second helices (kindly provided by Dr. A. Aguzzi, University Hospital of Zurich, Switzerland), or mAb 8H4, mapping epitopes around the 175–195 amino acid stretch (kindly provided by Dr. M.S. Sy, Case Western Reserve University, Cleveland, OH), in BSA (1%)-containing TBS (1∶5000), or PBS (1∶10000), respectively, and then rinsing (4 times) with the respective medium. (vi) Incubation with horseradish-peroxidase (HRP)-conjugated anti-mouse IgG secondary antibody (Santa Cruz Biotechnology) (1∶2000) in BSA (1%)-containing TBS (500 µl, 1 h, RT), or PBS, and then washing (4 times) with TBS, or PBS. (vii) Resuspension of pellets in 1 ml of TBS, or PBS, and sampling into 96 well-plates (200 µl/well, 3 wells/sample). (viii) Gravity-sedimentation of soil particles (10 min), careful removal of supernatants from each well, and resuspension of pellets in 75 µl/well of the ECL reagent (Pierce). (ix) Digitalisation (with a Kodak Imaging Workstation) of the resulting chemiluminescence, and densitometric analysis using the Kodak 1D software. Steps (i)-(iii) were omitted when assaying either PrP^C ^(i.e. not infected material), or rPrP. The whole procedure is accomplished in less than 6 hours.

### Cell culturing and scrapie cell assay (SCA)

The prion-susceptible mouse N2a-PK1 clonal cell line was kindly given by D-Gen Ltd. (London, UK), while the parental neuroblastoma N2a cell line was purchased from ATCC. Cells were routinely grown (37°C, humidified 5% CO_2_ atmosphere) in the OFCS medium (Optimem (Invitrogen) supplemented with fetal calf serum (10%), glutamine (2 mM), penicillin (100 U/ml) and streptomycin (100 µg/ml)). The day preceding the inoculation, cells were seeded onto 6 well-plates at a density of 2·10^5^ cells/well, while immediately before inoculation the growth medium was replaced with fresh OFCS medium (1 ml). Supernatants and pellets, obtained from soil-brain mixtures as described, were diluted in the OFCS medium (final volume, 1 ml), and then administered to cells. Following inoculation, cells were grown under the above conditions and splitted every 2–3 days into new 6 well-plates at the desired dilution (1∶3 or 1∶10), after detachment from the growth chamber and resuspension by gentle pipetting. After 4 to 10 passages, cells were processed by the Elispot assay. Appropriate controls were performed as reported under “Results and Discussion”. Given the tendency to lose competence for prion replication upon repeated passaging [30], inocula were never administered to N2a-PK1 cells that had been subjected to more than 5 passages. In all assays, each condition was run at least in duplicate. In our hands, the adequate amplification of soil-bound prions for the Elispot assay required less than 4 weeks.

### Elispot assay

The Elispot assay was performed according to [30] with minor modifications. (i) Elispot plates (Multiscreen HTS, Millipore) were activated by adding ethanol (50 µl, 70%) to each well, then rinsed twice with PBS (160 µl). (ii) In each used condition, cells were suspended in the OFCS medium (200 µl) and seeded (in duplicate, or triplicate) in the Elispot plates at a density of 3–6·10^4^ cells/well. Cells were adsorbed to the Immobilon-P membrane by suction, followed by drying of the plates (1 h, 50°C). (iii) Each well was incubated (90 min, 37°C) with 60 µl of a PK solution (1 µg/ml in the lysis buffer: 50 mM Tris-HCl (pH 8), 150 mM NaCl, 0.5% Na-deoxycholate, 0.5% Triton X-100), rinsed twice with PBS, treated with PMSF (1mM in PBS, 10 min, RT) and then with Tris-guanidinium thiocyanate (3M, 10 min, RT), and finally washed (4 times) in PBS. (iv) Each well, added with 120 µl of SuperBlock® (in TBS, 1 h, RT), was incubated (1 h, RT) firstly with 60 µl of mAb POM-1 (1∶5000), or 8H4 (1∶10000) (in TBS, containing 0.1% Tween-20 (TBST), and 1% non-fat dry milk) and washed (4 times) in TBST; then (1 h, RT) with 60 µl of alkaline phosphatase (AP)-conjugated anti-mouse IgG (in TBST) (1∶3000, Santa Cruz Biotechnology), and rinsed (4 times) with TBST. (vi) After removal of the plate's plastic underdrain, the exposed membrane was air-dried (RT) under a hood, and processed for colour development using an AP-substrate kit (Biorad), following the manufacturer's instructions. (vii) Digital images were collected through a high-resolution scanner (Epson). Occasionally, the resulting immunosignal was also densitometrically analysed.

## Supporting Information

Figure S1 Prions Are Adsorbed onto ASL Particles and Retain Infectivity. N2a-PK1 cells are inoculated with the supernatant (SN, upper lane), or pellet (sedimented fraction, PT, lower lane), derived from centrifuging healthy (hBH, columns 1 and 2), or RML-infected (columns 3 and 4), brain homogenates (2.5% (w/v)) incubated in the absence (-) (columns 1 and 3), or in the presence (+), of ASL (10 mg, columns 2 and 4) (see Materials and Methods). After 4 (1∶3) and 2 (1∶10) passages, cells are analysed for prion propagation by the Elispot assay (4·10^4^ cells/well). As shown, ASL addition to RML-infected homogenates causes a significant reduction, and a parallel increase, of infectivity in SN and PT fractions, respectively. This result clearly indicates that prions are sorbed onto soil particles, and remain infectious. The high-resolution image allows also to appreciate the presence of prion-infected single cell colonies. Conversely, no trace of infectivity is detected in hBH-treated samples. Other experimental details are as described in the legend to Fig. 2.
(0.45 MB PDF)

## References

[1] Prusiner SB (1998). Prions.. Proc Natl Acad Sci U S A.

[2] Collinge J (1999). Variant Creutzfeldt-Jakob disease.. Lancet.

[3] Belay ED, Maddox RA, Williams ES, Miller MW, Gambetti P (2004). Chronic wasting disease and potential transmission to humans.. Emerg Infect Dis.

[4] Sigurdson CJ, Aguzzi A (2007). Chronic wasting disease.. Biochim Biophys Acta.

[5] Mawhinney S, Pape WJ, Forster JE, Anderson CA, Bosque P (2006). Human prion disease and relative risk associated with chronic wasting disease.. Emerg Infect Dis.

[6] Hoinville LJ (1996). A review of the epidemiology of scrapie in sheep.. Rev Sci Tech.

[7] Miller MW, Williams ES (2003). Prion disease: horizontal prion transmission in mule deer.. Nature.

[8] Ryder S, Dexter G, Bellworthy S, Tongue S (2004). Demonstration of lateral transmission of scrapie between sheep kept under natural conditions using lymphoid tissue biopsy.. Res Vet Sci.

[9] Palsson PA, Prusiner SB, Hadlow WJ (1979). Rida (scrapie) in Iceland and its epidemiology.. Slow transmissible diseases of the nervous system.

[10] Fries GF (1996). Ingestion of sludge applied organic chemicals by animals.. Sci Total Environ.

[11] Hui CA (2004). Geophagy and potential contaminant exposure for terrestrial vertebrates.. Rev Environ Contam Toxicol.

[12] Miller MW, Williams ES, Hobbs NT, Wolfe LL (2004). Environmental sources of prion transmission in mule deer.. Emerg Infect Dis.

[13] Seeger H, Heikenwalder M, Zeller N, Kranich J, Schwarz P (2005). Coincident scrapie infection and nephritis lead to urinary prion excretion.. Science.

[14] Mathiason CK, Powers JG, Dahmes SJ, Osborn DA, Miller KV (2006). Infectious prions in the saliva and blood of deer with chronic wasting disease.. Science.

[15] Revault M, Quiquampoix H, Baron MH, Noinville S (2005). Fate of prions in soil: trapped conformation of full-length ovine prion protein induced by adsorption on clays.. Biochim Biophys Acta.

[16] Rigou P, Rezaei H, Grosclaude J, Staunton S, Quiquampoix H (2006). Fate of prions in soil: adsorption and extraction by electroelution of recombinant ovine prion protein from montmorillonite and natural soils.. Environ Sci Technol.

[17] Vasina EN, Dejardin P, Rezaei H, Grosclaude J, Quiquampoix H (2005). Fate of prions in soil: adsorption kinetics of recombinant unglycosylated ovine prion protein onto mica in laminar flow conditions and subsequent desorption.. Biomacromolecules.

[18] Cooke CM, Shaw G (2007). Fate of prions in soil: Longevity and migration of recPrP in soil columns.. Soil Biol Biochem.

[19] Rao MA, Russo F, Granata V, Berisio R, Zagari A (2007). Fate of prions in soil: Interaction of a recombinant ovine prion protein with synthetic humic-like mineral complexes.. Soil Biol Biochem.

[20] Leita L, Fornasier F, De Nobili M, Bertoli A, Genovesi S (2006). Interactions of prion proteins with soil.. Soil Biol Biochem.

[21] Johnson CJ, Phillips KE, Schramm PT, McKenzie D, Aiken JM (2006). Prions adhere to soil minerals and remain infectious.. PLoS Pathog.

[22] Cooke CM, Rodger J, Smith A, Fernie K, Shaw G (2007). Fate of prions in soil: detergent extraction of PrP from soils.. Environ Sci Technol.

[23] Ma X, Benson CH, McKenzie D, Aiken JM, Pedersen JA (2007). Adsorption of pathogenic prion protein to quartz sand.. Environ Sci Technol.

[24] Seidel B, Thomzig A, Buschmann A, Groschup MH, Peters R (2007). Scrapie agent strain 263K) can transmit disease via the oral route after persistence in soil over years.. PLoS ONE.

[25] Brown P, Gajdusek DC (1991). Survival of scrapie virus after 3 years' interment.. Lancet.

[26] Johnson CJ, Pedersen JA, Chappell RJ, McKenzie D, Aiken JM (2007). Oral transmissibility of prion disease is enhanced by binding to soil particles.. PLoS Pathog.

[27] Flechsig E, Hegyi I, Enari M, Schwarz P, Collinge J (2001). Transmission of scrapie by steel-surface-bound prions.. Mol Med.

[28] Caughey B, Raymond GJ (1993). Sulfated polyanion inhibition of scrapie-associated PrP accumulation in cultured cells.. J Virol.

[29] Weissmann C, Enari M, Klöhn PC, Rossi D, Flechsig E (2002). Transmission of prions.. Proc Natl Acad Sci U S A.

[30] Klöhn PC, Stoltze L, Flechsig E, Enari M, Weissmann C (2003). A quantitative, highly sensitive cell-based infectivity assay for mouse scrapie prions.. Proc Natl Acad Sci U S A.

[31] Wolf DC, Scott HD, Lavy TL, Dao TH (1989). Influence of sterilization methods on selected soil microbiological, physical, and chemical properties.. J Environ Qual.

[32] Shaw LJ, Beaton Y, Glover LA, Killham K, Meharg AA (1999). Re-inoculation of autoclaved soil as a non-sterile treatment for xenobiotic sorption and biodegradation studies.. Appl Soil Ecol.

[33] Connan H, Ray A, Thomas P, Guerbois J-P (2007). Effect of autoclaving temperature on calcium silicate-based building containing clay-brick waste.. J Therm Anal Calorim.

[34] Chung HH, Choi SW, Ok YS, Jung J (2004). EPR characterization of the catalytic activity of clays for PCE removal by gamma-radiation induced by acid and thermal treatments.. Chemosphere.

[35] Aguzzi A, Heikenwalder M (2006). Pathogenesis of prion diseases: current status and future outlook.. Nat Rev Microbiol.

[36] Brown P (1998). BSE: the final resting place.. Lancet.

[37] Hadlow WJ, Kennedy RC, Race RE (1982). Natural infection of Suffolk sheep with scrapie virus.. J Infect Dis.

[38] Sigurdson CJ, Williams ES, Miller MW, Spraker TR, O'Rourke KI (1999). Oral transmission and early lymphoid tropism of chronic wasting disease PrPres in mule deer fawns (Odocoileus hemionus).. J Gen Virol.

[39] Aringhieri R, Giachetti M (2001). Effect of sodium adsorption ratio and electrolyte concentrations on the saturated hydraulic conductivity of clay–sand mixtures.. Eur J of Soil Sci.

[40] Carter DL, Mortland MM, Kemper WD, Klute A, Page AL (1986). Specific surface.. Methods of soil analysis, part 1. Physical and mineralogical methods. 2nd ed.

[41] Brini M, Miuzzo M, Pierobon N, Negro A, Sorgato MC (2005). The prion protein and its paralogue Doppel affect calcium signaling in Chinese hamster ovary cells.. Mol Biol Cell.

