# Supplementary material for: Direct Detection of Soil-Bound Prions
Source: PLoS One. 2007 Oct 24;2(10):e1069. doi: 10.1371/journal.pone.0001069 (PMC2031919; doi:10.1371/journal.pone.0001069)
Supplement: Figure S1 — Prions Are Adsorbed onto ASL Particles and Retain Infectivity. N2a-PK1 cells are inoculated with the supernatant (SN, upper lane), or pellet (sedimented fraction, PT, lower lane), derived from centrifuging healthy (hBH, columns 1 and 2), or RML-infected (columns 3 and 4), brain homogenates (2.5% (w/v)) incubated in the absence (-) (columns 1 and 3), or in the presence (+), of ASL (10 mg, columns 2 and 4) (see Materials and Methods). After 4 (1∶3) and 2 (1∶10) passages, cells are analysed for prion propagation by the Elispot assay (4·104 cells/well). As shown, ASL addition to RML-infected homogenates causes a significant reduction, and a parallel increase, of infectivity in SN and PT fractions, respectively. This result clearly indicates that prions are sorbed onto soil particles, and remain infectious. The high-resolution image allows also to appreciate the presence of prion-infected single cell colonies. Conversely, no trace of infectivity is detected in hBH-treated samples. Other experimental details are as described in the legend to Fig. 2. (0.45 MB PDF) [file pone.0001069.s001.pdf]

Fig S1

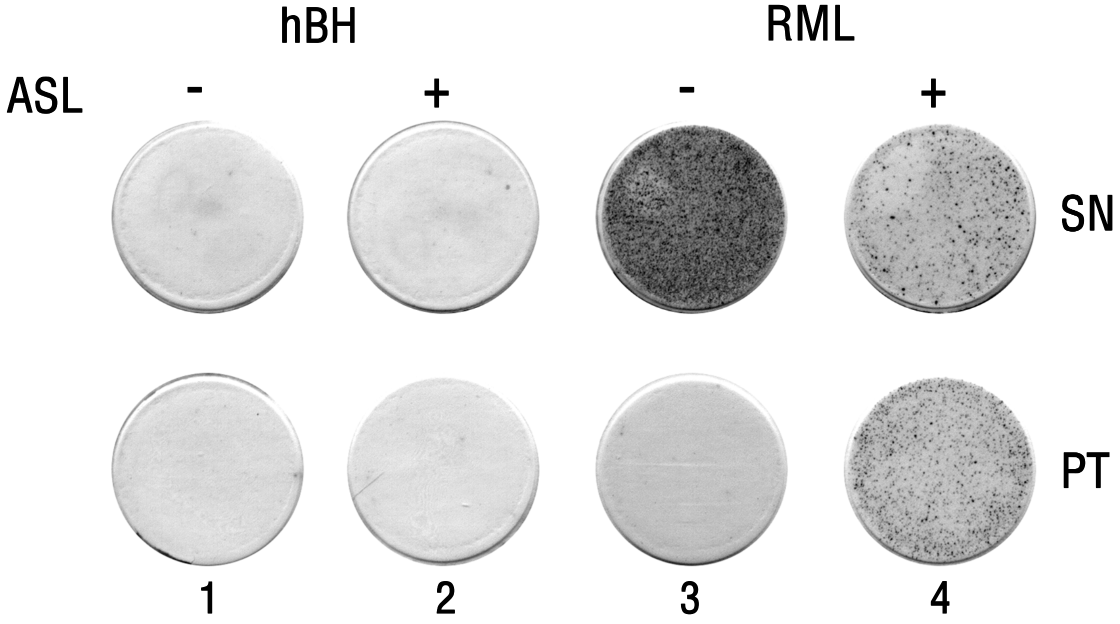

Figure S1. Prions Are Adsorbed onto ASL Particles and Retain Infectivity.

N2a-PK1 cells are inoculated with the supernatant (SN, upper lane), or pellet (sedimented fraction, PT, lower lane), derived from centrifuging healthy (hBH, columns 1 and 2), or RML-infected (columns 3 and 4), brain homogenates (2.5% (w/v)) incubated in the absence (-) (columns 1 and 3), or in the presence (+), of ASL (10 mg, columns 2 and 4) (see Materials and Methods). After 4 (1:3) and 2 (1:10) passages, cells are analysed for prion propagation by the Elispot assay ($4 \cdot 10^4$ cells/well). As shown, ASL addition to RML-infected homogenates causes a significant reduction, and a parallel increase, of infectivity in SN and PT fractions, respectively. This result clearly indicates that prions are sorbed onto soil particles, and remain infectious. The high-resolution image allows also to appreciate the presence of prion-infected single cell colonies. Conversely, no trace of infectivity is detected in hBH-treated samples. Other experimental details are as described in the legend to Fig. 2.
